# Age-related heterogeneity of type 1 diabetes mellitus in children: a single-center retrospective study

**DOI:** 10.1007/s12519-025-01004-3

**Published:** 2025-12-27

**Authors:** Shi-Yang Gao, Yi-Guo Huang, Li-Bo Wang, Qian-Wen Zhang, Guo-Ying Chang, Juan Li, Fei-Han Hu, Yu Ding, Xiu-Min Wang

**Affiliations:** Department of Endocrinology, Metabolism and Genetics, Shanghai Children’s Medical Center, Shanghai Jiao Tong University School of Medicine, DongFang Road, PuDong District, Shanghai, 200127 China

**Keywords:** Age of onset, Autoimmunity, Children, Islet cell function, Metabolism, Type 1 diabetes mellitus

## Abstract

**Background:**

Type 1 diabetes mellitus is a heterogeneous autoimmune disease with diverse characteristics between ethnicities and ages. Understanding this heterogeneity is essential for optimizing management and developing precision treatments. This study investigates the clinical, metabolic and immunological heterogeneity of type 1 diabetes mellitus in children across different ages of onset.

**Methods:**

A retrospective analysis of 401 children newly diagnosed with type 1 diabetes mellitus at a single center from January 2009 to August 2024 was conducted. We compared the clinical characteristics of our cohort with others from different countries. Patients were categorized into three age groups: 6 months–5 years, 5–10 years and ≥ 10 years at diagnosis. Clinical, metabolic, and immunological features were compared among groups.

**Results:**

The median cohort age was 8.58 years; 48.9% were male. Diabetic ketoacidosis occurred in 56.1% of patients, higher than in the Finnish and American cohorts. Most clinical characteristics are not significantly different among age groups. The 6 months–5 years group had a lower area under the curve (AUC) for C-peptide compared to the other age groups. The ≥ 10 years group was more likely to be thyroid antibody positive and have vitamin D deficiency. Immunologically, type 1 diabetes mellitus patients showed significantly increased counts of T lymphocytes, CD3 + CD8 + T cells and B lymphocytes, along with decreased interleukin-2 and increased interleukin-6 levels compared to healthy controls. Of note, the 6 months–5 years group had a higher CD4/CD8 ratio, which was negatively correlated with C-peptide AUC.

**Conclusions:**

Significant heterogeneity in type 1 diabetes mellitus features exists across age groups. Early-onset patients showed poorer islet function and late-onset patients were more prone to metabolic complications. Collectively our study emphasizes the need for age-specific management strategies.

**Graphical abstract:**

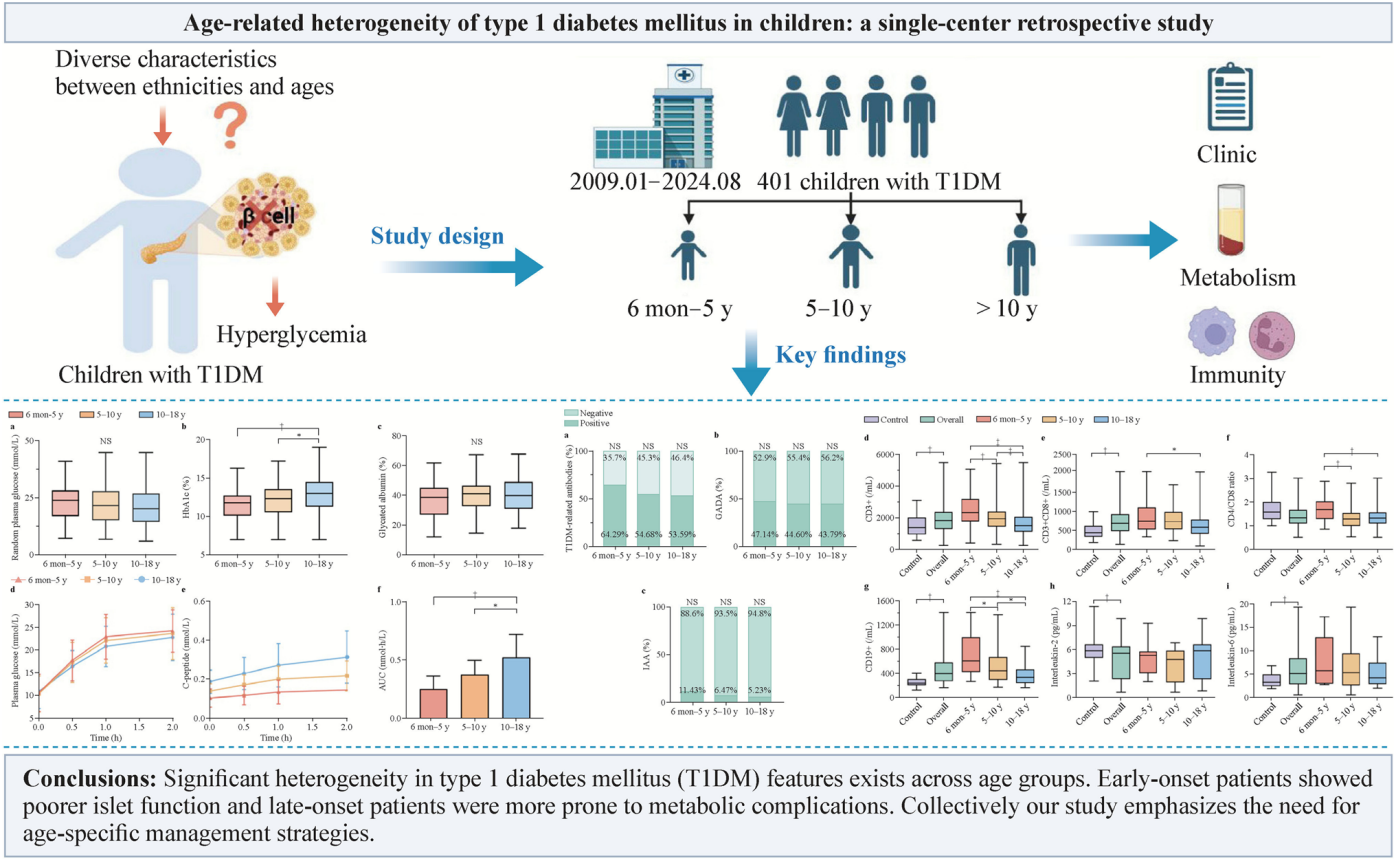

**Supplementary Information:**

The online version contains supplementary material available at 10.1007/s12519-025-01004-3.

## Introduction

Type 1 diabetes mellitus (T1DM) is one of the most common chronic autoimmune diseases in children, characterized by the destruction of pancreatic β-cells, islet dysfunction and absolute insulin deficiency [1]. Despite significant improvements in the survival rate of T1DM patients since the discovery of insulin over a century ago, prevention and treatment of this condition remain suboptimal [2]. In recent years, an increasing body of research has highlighted the highly heterogeneous nature of T1DM, not only in its clinical presentation but also in individual responses to therapeutic interventions [3]. This heterogeneity is closely associated with genetic factors, immune mechanisms, age at onset and environmental influences [4]. Clinically, children with early-onset T1DM often exhibit a more robust autoimmune response, a faster rate of β-cell destruction and a shorter honeymoon phase (a transient period characterized by a partial restoration of endogenous insulin secretion, leading to improved glycemic control and a significant reduction in exogenous insulin requirements) compared to those with later-onset disease, who typically have a relatively longer honeymoon period, different metabolic characteristics and insulin requirements [5]. In addition, racial and geographical factors play a crucial role in the heterogeneity of T1DM [6]. For example, Northern European countries, such as Finland and Sweden report the highest global incidence rates of T1DM, whereas Southern European countries, including Italy and Spain demonstrate significantly lower rates [7]. Similarly, in North America, T1DM incidence is markedly higher among Caucasians compared to African American and Latino populations; however, African Americans tend to have an earlier onset of the disease [8]. These findings underscore the intricate pathological mechanisms of T1DM across different onset ages, which are not adequately captured by current diabetes classification models. This limitation hinders effective clinical management strategies [9, 10]. According to World Health Organization DiaMond Project data (1985–1994), China had one of the lowest global incidences of T1DM in children (0.51 per 100,000 person-years) [11]. However, a recent nationwide study (2015–2019) revealed that the average annual clinical incidence among individuals aged 0–16 years had risen to 3.16 per 100,000, which included 6544 newly diagnosed patients, of whom 52.4% were female, 41.8% tested positive for T1DM-related antibodies and 52.7% presented with diabetic ketoacidosis (DKA) [12].

The present study aims to conduct a comprehensive retrospective analysis of the clinical, metabolic and immunological heterogeneity of children with T1DM across different onset ages in a Chinese cohort. By doing so, we seek to provide a theoretical foundation for individualized treatment and precision medicine at different ages of onset.

## Methods

### Patients

This is a single-center, retrospective cohort study conducted at a national-level pediatric medical center in a large city in China. The hospital serves as a regional pediatric medical center and admits patients from the city and more widely across the country. A total of 586 patients who were admitted to our hospital between January 2009 and August 2024 and were diagnosed with diabetes mellitus were approached. The selection of participants was based on strict inclusion and exclusion criteria to ensure data accuracy and consistency.

Inclusion criteria were as follows: (1) the diagnosis of T1DM was confirmed according to the American Diabetes Association’s Standards of Medical Care in Diabetes [13]. This requires the fulfillment of all four of the following criteria: low C-peptide levels; detection of at least one pancreatic islet autoantibody (whole-exome sequencing is required for antibody-negative individuals to exclude other diabetes types) along with at least one clinical criterion; acute onset with classic hyperglycemic symptoms (polyuria, polydipsia, polyphagia, or weight loss) potentially accompanied by ketosis/ketoacidosis; continuous insulin dependence since initial diagnosis; (2) aged between 6 months and 18 years, and (3) signed informed consent obtained from the participant’s legal guardian, with children over eight years old capable of comprehension personally signing informed consent forms.

Exclusion criteria included: (1) type 2 diabetes; (2) monogenic diabetes/syndromes; (3) secondary diabetes; (4) undefined diabetes; (5) non-newly diagnosed T1DM, and (6) no age record.

Clinical data for all patients were obtained from electronic medical records, including clinical and laboratory test results.

Legal guardian of all participants signed standard medical informed consent forms during their clinical visits, which included authorization for the use of treatment data in scientific research. In addition patients over eight years old also signed these consent forms. This study utilized only de-identified historical medical data without any additional interventions or patient contact. The study was approved by the Ethics Committee of our hospital (SCMCIRB-K2022007-1) and adhered to the ethical requirements of the Declaration of Helsinki.

### Clinical assessment

All participants underwent a comprehensive clinical assessment following their diagnosis of T1DM. Demographic information, including gender, age, medical history and family history was collected. According to the Chinese reference values released by the Chinese child growth standards (2009 edition) [14], age- and gender-independent height and body mass index (BMI) Z-scores were calculated. For children between 0 and 18 years old, overweight or obesity was also defined according to the Chinese reference values released by the Chinese child growth standards (2009 edition) [14]. Fasting venous blood samples were collected from all patients for analysis of plasma glucose, C-peptide, glycated hemoglobin (HbA1c), pH, ketone bodies and T1DM-related antibodies. Specifically these included glutamic acid decarboxylase antibody (GADA), insulinoma antigen-2 antibody (IA-2A), insulin autoantibodies (IAA) and islet cell antibody (ICA). Additional tests included vitamin D levels (*n* = 249), thyroid antibodies (*n* = 391), cytokine levels and lymphocyte subsets (*n* = 80). All of the tests were processed in our hospital. Analysis of C-peptide, HbA1c, ketone bodies and autoantibodies used centrifuged samples and electrochemiluminescence immunoassays or enzyme-linked immunosorbent assays with commercial kits. Serum samples were centrifuged prior to analysis. Vitamin D and cytokine levels were quantified using chemiluminescence immunoassay and multiplex bead-based assays, respectively. Vitamin D deficiency was defined as a serum 25-hydroxyvitamin D level < 20 ng/mL, and insufficiency as a level of 20–29 ng/mL [15]. Thyroid antibodies were measured via immunochemiluminescence and lymphocyte subsets were analyzed using flow cytometry. Urine was tested for glucose and ketones by enzymatic strips and confirmed spectrophotometrically when necessary. For participants diagnosed with T1DM, the diagnosis of DKA was made based on the International Society for Pediatric and Adolescent Diabetes guidelines, which includes hyperglycemia (blood glucose ≥ 11 mmol/L), metabolic acidosis (venous blood pH < 7.3 or serum bicarbonate < 15 mmol/L) and the presence of ketone bodies in blood or urine [16]. In addition, an oral glucose tolerance test (OGTT) was performed on some patients; this was performed on an empty stomach in the morning. For patients presenting with hyperglycemia or DKA, some specific tests (C-peptide, antibodies, thyroid function, cytokines and vitamin D) were collected after clinical stabilization and glycemic control. We also recorded insulin treatment modalities (such as continuous subcutaneous insulin infusion or multiple daily subcutaneous injections), daily insulin dosage, length of hospital stay, and hospitalization costs. All data were systematically collected and organized through the electronic medical record system for further analysis.

### Patient grouping

Children newly diagnosed with T1DM were categorized into three age groups based on their age at onset: 6 months–5 years, 5–10 years and ≥ 10 years. Clinical characteristics, metabolic indicators and immunological features were systematically compared across these age groups to identify any differences and patterns.

### Statistical analysis

Categorical data were expressed as frequencies (%) and analyzed using Cochran-Armitage trend Chi-square test for ordered group comparisons, Chi-squared or Fisher’s exact test for non-trend analyses [applied when > 20% of expected frequencies were < 5 or any expected frequency was zero (expected frequencies = (row total × column total)/grand total under null hypothesis)]. Continuous data were assessed for distribution properties using Shapiro–Wilk normality tests and Levene’s variance homogeneity tests. Data were presented as mean ± standard deviation (SD) for normally distributed variables or median, 25th percentile, 75th percentile (P25, P75) for non-normal distributions. Monotonic trends across ordered age groups (6 months–5 years, 5–10 years, 10–18 years) were evaluated via analysis of covariance (ANCOVA) trend tests using age midpoints (2.5, 7.5, 14 years). Group comparisons employed: Kruskal–Wallis test for non-normal data and Welch’s analysis of variance (ANOVA) for normal but heteroscedastic data. When omnibus tests reached significance, post hoc pairwise comparisons were conducted (Dunn’s test), with all *P* values adjusted for multiplicity using the Benjamini–Hochberg method. Adjusted *P* < 0.05 was considered statistically significant. OGTT variables were analyzed using repeated-measures analysis of variance (Repeated Measures ANOVA), with group as the between-subjects factor and time point as the within-subjects factor. Sphericity was assessed using Mauchly’s Test, with Greenhouse–Geisser correction applied when the assumption was violated. The analysis examined the main effects of time, main effects of group and time-by-group interaction effects.

All statistical analyses were conducted using SPSS version 25.0 (Statistical Package for the Social Sciences Inc., Chicago, IL, USA). A two-tailed *P* value of < 0.05 was considered statistically significant.

## Results

### Demographics and clinical data

**Fig. 1:**
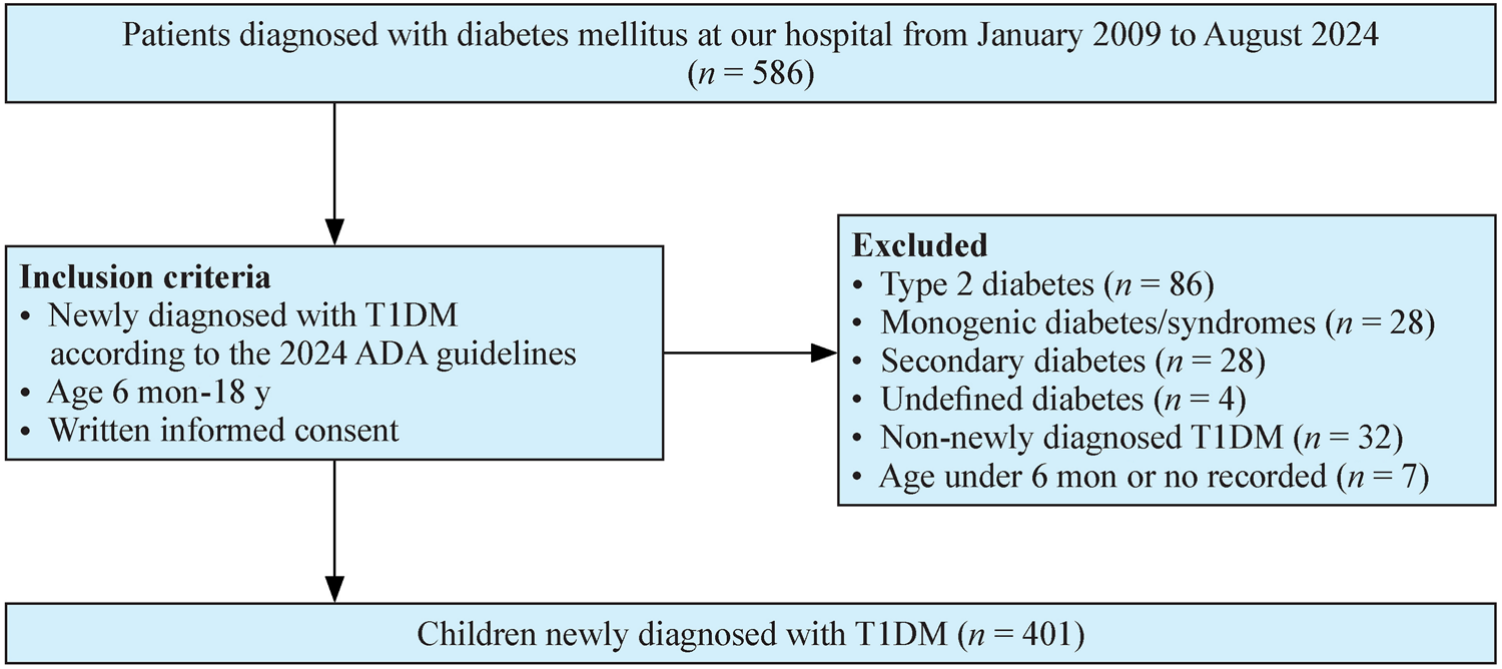
Flow chart of patient selection process. *T1DM* type 1 diabetes; *ADA* American Diabetes Association

Between January 2009 and August 2024, a total of 586 patients admitted to our hospital were diagnosed with diabetes mellitus. Of these, 185 patients were excluded from the study: 86 with type 2 diabetes (T2DM), 28 with monogenic diabetes/syndromes, 28 with secondary diabetes and four with unclassified diabetes. In addition, 32 patients with a previous diagnosis of T1DM and seven patients with missing age records were excluded. Ultimately, 401 newly diagnosed T1DM patients under 18 years of age were included in the study (Fig. 1), comprising 196 males and 205 females. All patients fulfilled our diagnostic criteria: the C-peptide levels were low when their conditions were stable; 224 (55.9%) patients were antibody-positive, while the 177 antibody-negative cases underwent whole-exome sequencing to exclude other diabetes types; all patients presented with classic symptoms; all received insulin therapy during hospitalization and were insulin-dependent, though 20 later discontinued treatment post-discharge due to non-adherence. The majority of patients were from the Eastern regions of China, with an average age of 8.58 ± 3.69 years. Only 1.2% (4/341) of patients had a family history of T1DM. A history of preceding infections was reported in 17.8% of patients, with respiratory tract infections being the most common (16.0%). DKA was the initial presentation in 56.1% of patients. Median random blood glucose level was 23.15 (17.38–29.00) mmol/L and the HbA1c level was 12.5% (10.6%–13.9%). At the time of diagnosis, 8.1% (29/357) of patients had positive urinary protein tests and 29.5% (81/275) showed elevated urinary microalbumin levels. Following effective blood glucose control, most patients experienced a reversal of proteinuria and urinary microalbumin levels decreased. Among the 138 patients who underwent ophthalmological examinations, no retinopathy was observed. However, two patients had conjunctivitis, one had corneal epithelial damage, one had cataracts and one had elevated intraocular pressure. Given the young age and short disease duration of the study cohort, no microvascular or macrovascular complications were observed. All newly diagnosed T1DM patients received insulin therapy during their hospital stay. Median length of hospitalization was 8 (6–10) days, with a median hospitalization cost of 10,609 (6815–16,354) CNY. Among the 381 patients who continued insulin therapy after discharge, only 6.3% used continuous subcutaneous insulin infusion; the remaining patients were treated with multiple daily subcutaneous insulin injections.

### Comparison between our cohort and cohorts from other countries

To explore racial differences in the clinical characteristics of children with T1DM, we compared the features of our cohort with those from other countries: Finland [17], United States [18], and India [19]. The specific characteristics of participants from all four cohorts are presented in Table 1. The age of onset for patients in our cohort was younger than that observed in the cohorts from the United States and India. With respect to gender distribution, the proportion of males in our cohort was 48.9% of patients (Finland: 58.3%; United States: 56%; India: 53.1%).The incidence of DKA tended to be higher in our cohort compared to the Finnish and USA cohorts but was lower than in the Indian cohort. With respect to T1DM-related islet autoantibody, the positivity rate of GADA in our cohort was 46.8%; this was lower than the 72.98% reported in the Finnish cohort.

**Table 1:** Clinical features of our cohort compared with Finland, or United States or Indian study

Features	Our cohort (*n* = 401)	Finland cohort (*n* = 785)	United States cohort (*n* = 389)	Indian cohort (*n* = 535)
Age at diagnosis, y	8.58 ± 3.69	8.41 ± 3.75	9.6 ± 0.2	12.0 ± 5.4
Boys, *n* (%)	196 (48.9)	458 (58.3)	218 (56.0)	284 (53.1)
Family history of T1DM, *n* (%)	4 (1.2)	89 (11.3)	NA	119 (22.2)*
BMI Z-score	− 0.108 ± 1.02	− 0.19	0.1 (− 1.1, 1.2)	NA
Ketoacidosis, *n* (%)	225 (56.1)	228 (30.8)	188 (48.3)	500 (93.5)
pH	7.32 (7.11‒7.39)	7.36	7.32 (7.18‒7.38)	NA
Plasma glucose, mmol/L	23.15 (17.38‒29.00)	22.8	NA	NA
HbA1c, %	12.5 (10.6–13.9)	11.1	11.9 ± 2.5	9.3 ± 2.3
Total autoantibodies (+), *n* (%)	224 (55.9)	562 (96.7)	NA	NA
IA-2A (+), *n* (%)	89 (24.9)	432 (74.4)	NA	24 (34.3)
GADA (+), *n* (%)	167 (46.8)	424 (73.0)	NA	49 (72.1)

^*^ *P* < 0.05. Data are presented as *n* (%), mean ± SD or median (P25, P75)

*T1DM* type 1 diabetes mellitus; *BMI* body mass index; *HbA1c* glycated hemoglobin; *pH* potential of hydrogen; *IA-2A* insulinoma antigen-2 antibody; *GADA* glutamic acid decarboxylase antibody; *NA* data not available; *SD* standard deviation; *P25* the 25th percentile; *P75* the 75th percentile

### Comparative analysis by age

To further investigate heterogeneity in clinical characteristics, metabolic function, and immunological features in children with T1DM across different age groups, we divided the patients into three groups based on their age at diagnosis: the 6 months–5 years group (*n* = 74, 18.5%), the 5–10 years group (*n* = 155, 38.7%), and the 10–18 years group (*n* = 172, 42.9%).

#### Clinical characteristics across age groups

Comparison of clinical characteristics across the three age groups is summarized in Table 2. Height and BMI Z-scores in all three groups were below the normal values for children of the same age and gender; differences among the groups were not statistically significant. Regarding family history of T2DM, trend testing revealed a significantly increasing trend with age. This is likely attributable to the older age of relatives in the adolescent group; age is a predominant risk factor for the development of T2DM. The average incidence of DKA across the three groups was 56.1%, with no statistically significant differences observed between the groups. The 6 months–5 years group had a significantly longer hospital stay compared to the other two groups and hospitalization costs were higher. In terms of treatment modalities, the proportion of patients using continuous subcutaneous insulin infusion therapy was slightly higher in the 6 months–5 years group compared to the 5–10 years group, but this difference did not reach statistical significance.

**Table 2:** Clinical characteristics among the age groups

Characteristics	Groups			*P* for trend
	6 mon–5 y (*n* = 74)	5–10 y (*n* = 155)	10–18 y (*n* = 172)	
Age at diagnosis, y	3.06 ± 1.21	7.35 ± 1.45	12.11 ± 1.53	
Boys, *n* (%)	41 (55.4)	67 (43.2)	88 (51.2)	NS
Overweight or obesity, *n* (%)	10 (13.5)	19 (12.3)	30 (17.4)	NS
BMI Z-score	− 0.29 ± 0.86	− 0.31 ± 0.87	− 0.34 ± 1.08	NS
HA Z-score	0.21 ± 1.26	0.26 ± 1.41	0.34 ± 1.95	NS
Family history of T1DM, *n* (%)	0 (0)	2 (1.5)	2 (1.4)	NS
Family history of T2DM, *n* (%)	10/61 (16.4)	46/132 (36.4)	61/148 (42.6)	< 0.01*
Ketoacidosis, *n* (%)	41 (56.9)	89 (59.7)	100 (58.1)	NS
Average length of stay, d	10 (6–14)	9 (7–10)	7 (5–10)	< 0.01*
CSII, *n* (%)	7 (10.5)	5 (3.5)	12 (7.0)	NS
Insulin dosage, iu/kg/d	0.67 ± 0.29	0.68 ± 0.23	0.71 ± 0.23	NS

^*^P < 0.01. Data are presented as *n* (%), mean ± SD, or median (P25, P75)

The insulin dose and CSII rates presented were collected at the time of discharge. Categorical data were analyzed using the Cochran-Armitage trend chi-square test while quantitative data were examined through ANCOVA-based trend analysis with covariate adjustment for linear trend testing

*T1DM* type 1 diabetes mellitus; *T2DM* type 2 diabetes mellitus; *BMI* body mass index; *BMI Z* body mass index Z-score; *HA Z* height-for-age Z-score; *CNY* Chinese Yuan; *CSII* continuous subcutaneous insulin infusion; *NS* not significant; *ANOVA* analysis of variance; *P25* the 25th percentile; *P75* the 75th percentile

#### Glycemic and β-cell function across age groups

**Fig. 2:**
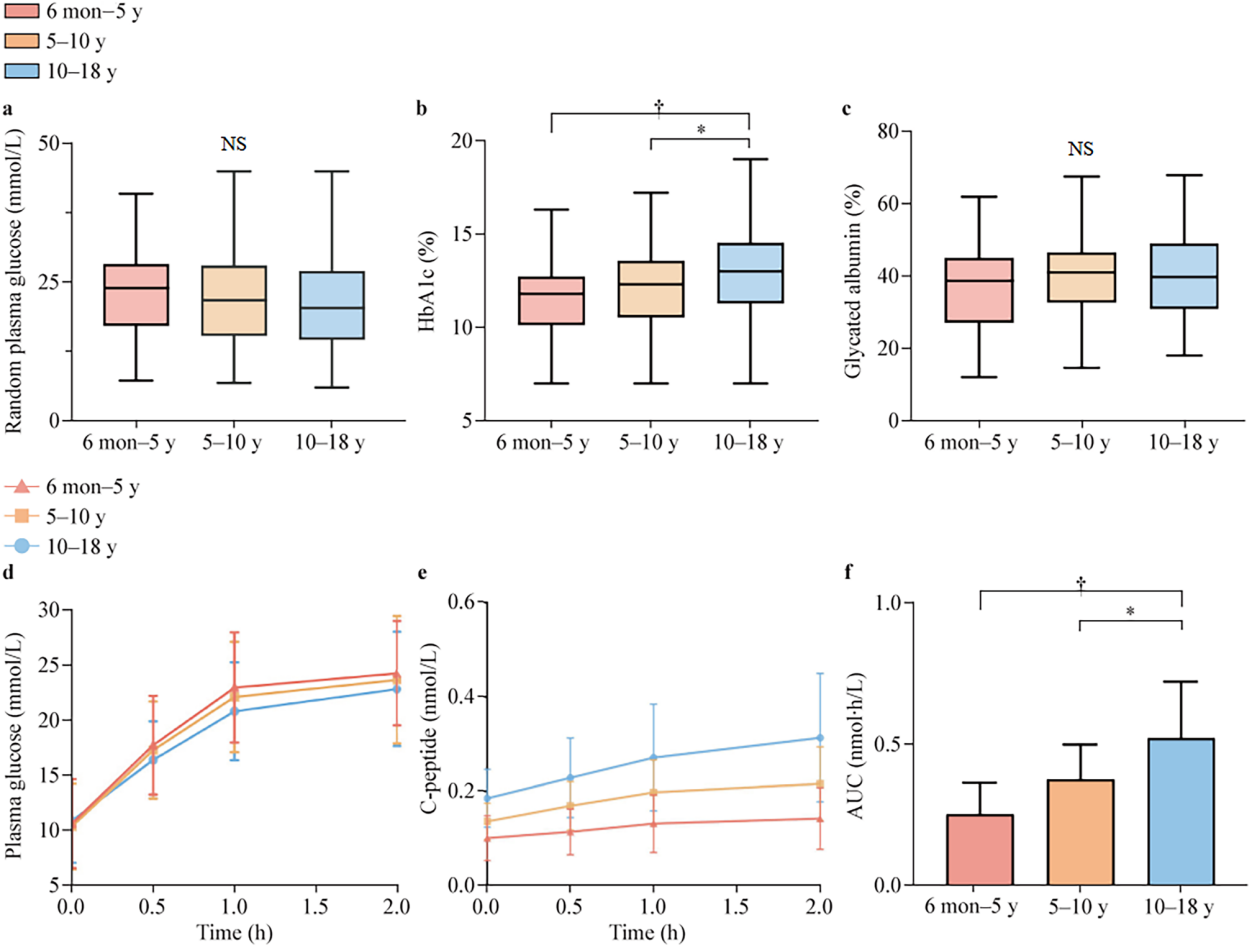
Blood glucose and β-cell function in children with T1DM across three age groups. **a** Random plasma glucose, **b** HbA1c, **c** glycated albumin, **d** glucose response to an OGTT. Statistical significance from repeated-measures ANOVA: main effect of time (*P* < 0.001), time × group interaction (*P* = 0.008). Quadratic trend difference between six months and five years group and others: *F* = 5.64, *P* = 0.004. **e** C-peptide secretion during OGTT, **f** area under the C-peptide release curve. Significant differences between age groups are indicated with *P < 0.05, †*P* < 0.001. *T1DM* type 1 diabetes; *OGTT* oral glucose tolerance test; *ANOVA* analysis of variance

Comparison of blood glucose levels and pancreatic β-cell function across the three age groups is illustrated in Fig. 2, with more detail provided in Supplementary Table 1. At diagnosis, the median HbA1c level for all patients was 12.5%. The HbA1c level in the 6 months–5 years group was significantly lower than in the 5–10 years group and the 10–18 years group. While there were no significant differences in fasting blood glucose levels among the three groups, fasting C-peptide levels showed a marked age-related difference. The 6 months–5 years group had significantly lower fasting C-peptide levels compared to the 10–18 years group. Among the 145 patients who completed the OGTT, distinct patterns of glucose and C-peptide responses were observed across the age groups. The youngest group (6 months–5 years) had the most rapid and extreme glucose spike after intake. The C-peptide response was attenuated and delayed in all groups, with no age-related differences. However, the oldest age group (10–18 years) consistently exhibited higher C-peptide levels at baseline, 30 minutes and 1 hour compared to the other two groups. Moreover, the area under the curve (AUC) of C-peptide differed significantly among the three groups. AUC in the 10–18 years group was significantly higher than the other two groups further confirming the heterogeneity in pancreatic β-cell function among T1DM patients of different ages.

#### Metabolism across age groups

The metabolic characteristics across the three age groups are presented in Supplementary Table 2. The number of thyroid antibody positive patients increased with age, with the 10–18 years group showing a significantly higher proportion (21.4%) compared to the 6 months–5 years group (3.4%) and the 5–10 years group (6.7%). In our cohort, vitamin D levels were 26.42 (21.15–32.31) ng/mL in children aged 6 months–5 years, 16.09 (12.34–22.19) ng/mL in those aged 5–10 years and 14.90 (10.20–19.69) ng/mL in those aged 10–18 years. The prevalence of both vitamin D insufficiency and deficiency was significantly higher in the older age groups (5–10 years and 10–18 years) compared to the youngest group (6 months–5 years).

#### Diabetes-related antibodies and immune function across age groups

**Fig. 3:**
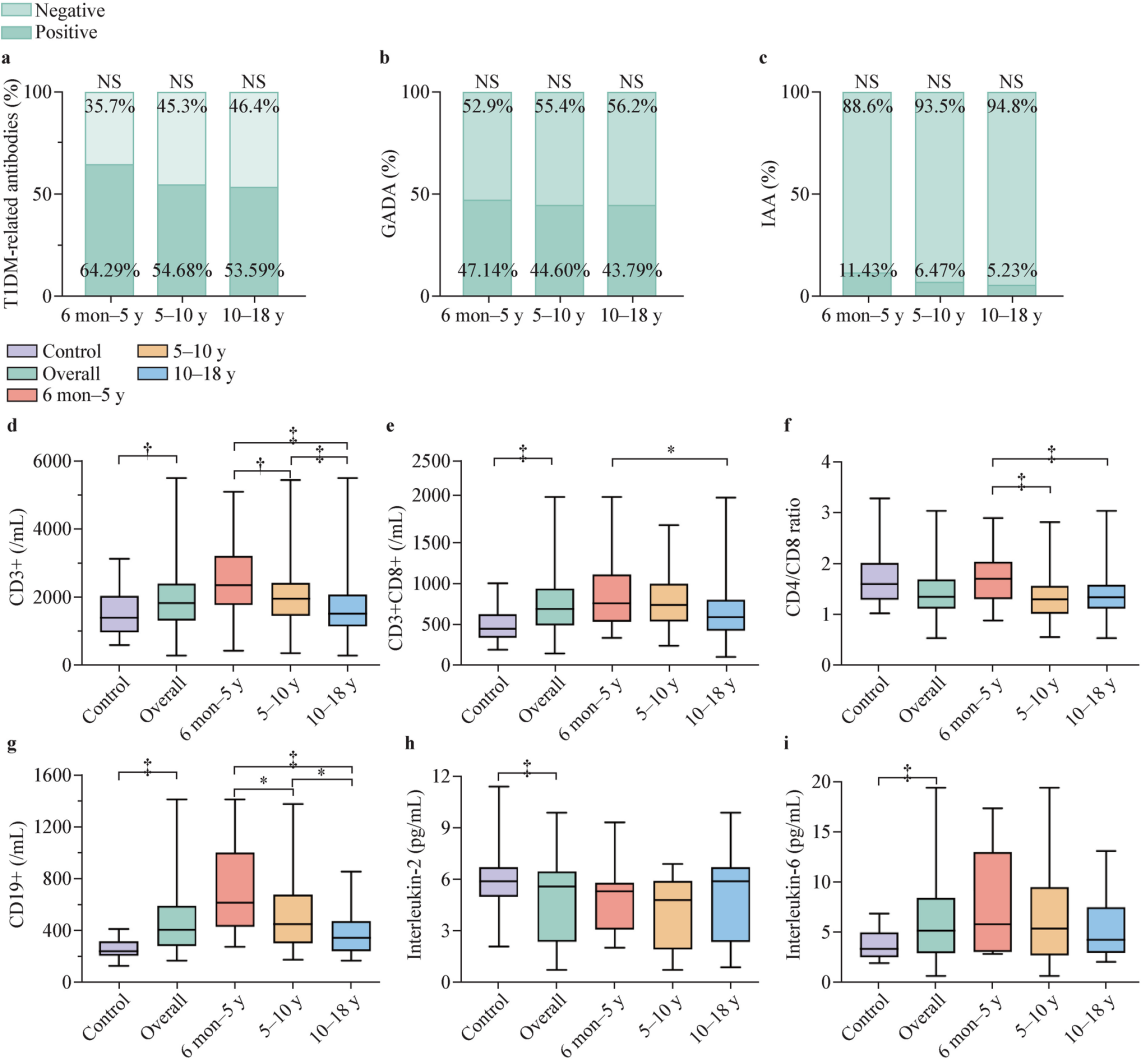
Diabetes-related antibodies and immune function among the age groups. **a** T1DM-related antibodies, **b** GADA, **c** IAA, **d** CD3 + T cells, **e** CD3 + CD8 + T cells, **f** CD4/CD8, **g** CD19 + lymphocyte, **h** IL-2, **i** IL-6. *P < 0.05, †*P* < 0.01, ‡*P* < 0.001. *T1DM* type 1 diabetes; *GADA* glutamic acid decarboxylase antibody; *IAA* insulin auto antibodies; *IL* interleukin; *AUC* area under curve

Positive antibody tests did not differ by age, with GADA being the most frequently detected at 46.8%, followed by IA-2A at 24.9%, ICA at 21.3% and IAA at 7.0%. (Fig. 3, Supplementary Table 3).

To evaluate immune function in patients with T1DM we selected 86 healthy children to act as a control group. We compared immune function and cytokine levels between the two groups and further analyzed immune differences among T1DM patients of different age groups (Fig. 3). The average age of healthy children was 7.73 ± 4.39 years; this did not differ significantly from T1DM patients. In a comparison of lymphocyte subsets, T1DM patients had a significantly higher count of CD3 + lymphocytes compared to the control group; CD3 + CD8 + lymphocytes were also increased. Furthermore, the CD19 + lymphocyte count was significantly elevated in T1DM patients compared to healthy controls suggesting that B lymphocytes may also play an important role in the pathogenesis of T1DM. Immune function varied significantly among the different age groups in T1DM patients. The 6 months–5 years group exhibited a significantly higher CD3 + lymphocytes count compared to the 5–10 years group and the 10–18 years group. In addition, the CD4/CD8 was significantly higher in the 6 months–5 years group, indicating a more robust immune response in early-onset patients. There was a significant negative correlation between the CD4/CD8 and the C-peptide AUC (*r* =  − 0.231, *P* < 0.05); a higher CD4/CD8 may be associated with more severe impairment of pancreatic β-cell function. With respect to cytokine levels, T1DM patients had significantly lower levels of interleukin-2 (IL-2) than healthy controls, while interleukin-6 (IL-6) levels were significantly elevated. These data indicate enhanced pro-inflammatory and reduced anti-inflammatory activity in T1DM patients. No significant differences in cytokine levels were observed among the different age groups (Supplementary Table 4).

## Discussion

This study explored the heterogeneity in clinical, metabolic and immunological characteristics among children with T1DM across different ethnicities and ages of onset. Clinical features in our cohort showed variations from other populations, which may be attributed to a combination of genetic, environmental and socio-cultural factors [20, 21]. First, the gender distribution in our cohort was relatively balanced, in contrast to the Finnish and United States cohorts where males were numerically predominant. This observation aligns with previous studies showing a higher incidence of T1DM in Nordic countries, particularly among boys and may be related to genetic variations, such as HLA gene subtypes [1, 4]. Moreover, the prevalence of a family history of T1DM in our cohort tended to be lower than in the Finnish and Indian cohorts, reflecting differences in genetic susceptibility across ethnic groups [4]. Second, significant disparities in immune responses and autoantibody expression have been documented among T1DM patients of diverse racial backgrounds [22, 23]. The antibody positive rates for T1DM-related antibodies in our cohort were different from those reported in Finland. Antibody-negative patients were defined based on clinical manifestations and laboratory investigations, with monogenic diabetes excluded by whole-exome sequencing. Notably, the antibody positive rate of 41.8% reported in the Chinese pediatric T1DM cohort further substantiates divergent immunopathology in Asian populations [12], mechanistically anchored to HLA haplotype disparities. Children in our cohort had higher HbA1c levels and a higher incidence of DKA (56.1%) compared to the Finnish and United States cohorts. This is closely associated with poor early recognition of childhood T1DM in primary healthcare settings in China, leading to a significantly increased risk of acute complications [24]. As our hospital is a pediatric institution treating patients aged 0–18 years, age differences across cohorts (Finland: < 15 years; USA/India: < 20 years) may partially explain the observed transnational heterogeneity in clinical characteristics.

Substantial heterogeneity was also observed in the clinical characteristics of children with T1DM across different age groups at onset. Early-onset (6 months–5 years) T1DM patients required significantly longer hospitalization than older age groups, reflecting the greater disease management challenges in young children.[25]. In terms of pancreatic β-cell function, distinct glucose change trends were observed after glucose intake across the three age groups, with the 6 months to 5 years group demonstrating a more pronounced rise in glucose. The C-peptide AUC was significantly lower in the 6 months–5 years group. This is consistent with previous research showing that early-onset T1DM is associated with a more aggressive decline in β-cell function. It also emphasizes the need for more intensive interventions to protect residual β-cell function and slow disease progression in early-onset patients [26]. Children with later-onset T1DM may experience a longer "honeymoon phase," during which residual β-cell function is partially preserved shortly after diagnosis [27]. Although patients with late-onset T1DM (≥ 10 years) retained relatively better β-cell function, they showed higher HbA1c levels. This suggests that disease onset was more insidious or that a longer time elapsed between onset and diagnosis.

The thyroid autoantibody positive rate increased with age in our cohort, reinforcing the well-established association between T1DM and autoimmune thyroid disease (AITD) [28, 29]. This finding underscores the importance of routine monitoring of thyroid function and autoantibodies in the management of T1DM, particularly during puberty and in patients with a family history of thyroid disorders. Consistent with previous studies [21, 30], our cohort exhibited lower vitamin D levels compared to healthy controls [31]. A large study of 11,116 Chinese children reported median vitamin D levels of 25.41, 20.92, and 19.18 ng/mL in preschool, school-aged and adolescents, respectively. Vitamin D levels were lower in older age groups in our study, a pattern that was also observed in the healthy group; this represents a general age-related trend [32, 33]. Given its immunomodulatory role and association with disease progression [34, 35], vitamin D deficiency may exacerbate immune-metabolic dysfunction in T1DM, underscoring the need for monitoring and supplementation, particularly in older children. Vitamin D supplementation could be a valuable adjunctive treatment strategy for patients with T1DM to improve immune function and reduce the risk of complications.

Children with T1DM showed immune system activation [36]. Significantly increased IL-6 levels in T1DM patients, a crucial pro-inflammatory cytokine associated with β-cell destruction, reveal a heightened inflammatory response in these children [37]. IL-2, critical for regulator T cell (Treg) differentiation and immune suppression, was reduced impairing regulatory function and exacerbating β-cell destruction [38, 39]. These findings highlight the central role of inflammation in the pathogenesis of T1DM [40], underscoring the therapeutic potential of targeting pro-inflammatory pathways or restoring IL-2 to modulate immune activity [41]. Consistent with a prior study [42], the 6 month–5 year group exhibited a significantly higher CD4/CD8 ratio, which correlated negatively with C-peptide AUC, indicating greater impairment of β-cell function. The greater immune system activity in young children may intensify the autoimmune attack, accelerating β-cell loss [43]. A deeper understanding of immunological changes as a function of age could provide essential insights for developing more personalized treatment strategies for T1DM patients across different ages.

This study has several limitations. First, although a large number of local and national patients were admitted to our hospital, the existence of other pediatric medical institutions in the city has the potential for diversity of newly diagnosed diabetic children. Therefore, there may be some selection bias. Future studies need to be conducted in multiple centers to obtain more representative samples. Second, the size of the healthy control group was relatively small (*n* = 86), which may limit generalizability. Third, the lack of long-term follow-up data restricts our analysis to cross-sectional clinical and immunological data. Future studies should focus on long-term follow-up to gain a comprehensive understanding of the prognostic differences between early and late-onset T1DM patients.

In conclusion, this study demonstrates age-stratified heterogeneity in pediatric T1DM, characterized by the prevalence of thyroid autoantibody positivity and vitamin D deficiency progressively increasing with age and being associated with immune dysregulation. Early-onset patients show rapid β-cell decline with elevated CD4/CD8 ratios, while late-onset patients retain greater β-cell function but face increased metabolic risks. These findings contribute to the development of precise treatment strategies tailored to different ages of T1DM patients.

## Supplementary Information

Below is the link to the electronic supplementary material.

Supplementary file1 (DOCX 28 KB)

## Data Availability

The datasets generated and/or analyzed during the current study are not publicly available but are available from the corresponding author on reasonable request.
