# Supplementary material for: Age-related heterogeneity of type 1 diabetes mellitus in children: a single-center retrospective study
Source: World J Pediatr. 2025 Dec 27;22(2):247–57. doi: 10.1007/s12519-025-01004-3 (PMC12923387; doi:10.1007/s12519-025-01004-3)
Supplement: Supplementary file 1 — Supplementary file1 (DOCX 28 KB) [file 12519_2025_1004_MOESM1_ESM.docx]

**Supplementary material (SM)**

**Heterogeneity in 401 Children with Type 1 Diabetes Mellitus Across Age Groups: A Single-Center Retrospective Study**

SM Table 1. Glycemic and β-cell Function Among the age Groups

| Characteristic | Groups | | | *p* for trend | | |
| --- | --- | --- | --- | --- | --- | --- |
|  | 6m–5 years  （n=74） | 5–10 years  （n=155） | 10–18 years  （n=172） | 6m–5 years  vs 5–10 years | 6m–5 years vs 10–18 years | 5–10 years vs 10–18 years |
| Random Plasma Glucose, mmol/L | 24.0（17.33-27.58） | 21.80（15.58-27.85） | 20.4（14.85-27.0） | NS | | |
| HbA1c（%） | 11.8（10.2-12.7） | 12.3（10.5-13.5） | 12.9（11.2-14.5） | NS | **<0.001***** | **<0.05*** |
| Glycated albumin （%） | 38.7（27.48-45.0） | 41.0（33.4-46.0） | 40.38±11.52 | NS | | |
| Plasma Glucose, mmol/L | 12.25（9.05-14.73） | 10.1（8.7-15.3） | 11.3（8.45-14.7） | NS | | |
| AUC, nmol*h/L | 0.25±0.11 | 0.37±012 | 0.52±0.21 | NS | **<0.001********* | **<0.05*** |

SM Table 2. Metabolism Among the age Groups

| Characteristic | Groups | | | *p* for trend | | |
| --- | --- | --- | --- | --- | --- | --- |
|  | 6m–5 years  （n=74） | 5–10 years  （n=155） | 10–18 years  （n=172） | 6m–5 years  vs 5–10 years | 6m–5 years vs 10–18 years | 5–10 years vs 10–18 years |
| FT4, pmol/l | 11.04（9.48-13.13） | 13.43（11.67-15.64） | 13.21（11.57-15.59） | **<0.001***** | **0.001***** | NS |
| TSH, uIU/ml | 1.48（1.06-1.86） | 1.96（1.33-2.83） | 1.67（0.93-2.21） | NS | | |
| vitamin D, ng/ml | 26.42 (21.15-32.31) | 16.09 (12.34-22.19) | 14.90 (10.20-19.69) | **<0.001***** | **<0.001***** | **<0.05*** |
| Vitamin D Insufficient, n (%) | 43.18% | 26.26% | 15.09% | **<0.001***** | **<0.001***** | NS |
| vitamin D deficiency, n (%) | 25.00% | 66.67% | 75.47% | **<0.001***** | **<0.001***** | NS |

SM Table3. Diabetes-related antibodies Among the age Groups

| Characteristic | Groups | | | *p* for trend | | |
| --- | --- | --- | --- | --- | --- | --- |
|  | 6m–5 years  （n=74） | 5–10 years  （n=155） | 10–18 years  （n=172） | 6m–5 years  vs 5–10 years | 6m–5 years vs 10–18 years | 5–10 years vs 10–18 years |
| T1DM-related antibodies, n (%) | 64.29% | 54.68% | 53.59% | NS | NS | NS |
| GADA, n (%) | 47.14% | 44.60% | 43.79% | NS | NS | NS |
| IA-2A, n (%) | 22.86% | 28.78% | 21.57% | NS | NS | NS |
| ICA, n (%) | 25.71% | 22.30% | 17.65% | NS | NS | NS |
| IAA, n (%) | 11.43% | 6.47% | 5.23% | NS | NS | NS |

SM Table4. Immune function Among the age Groups

| Characteristic | Control | Overall | P | 6m–5 years  （n=18） | 5–10 years  （n=25） | 10–18 years  （n=37） | P |
| --- | --- | --- | --- | --- | --- | --- | --- |
| CD3+#, n/ul | 1390.70（984.49，1992.73） | 1823.31（1322.05，2381.79） | <0.01** | 2351.15（1776.44，3163.77） | 1952.46（1466.48，2408.46） | 1510.71（1146.89，2058.66） | <0.001*** |
| CD3+CD8+#,n/ul | 453.24（347.0，628.12） | 696.98（499.06，945.37） | <0.001*** | 767.32（544.75，1115.08） | 746.98（551.7，1001.91） | 596.02（441.52，805.03） | <0.05* |
| CD4/CD8 | 1.60（1.30，2.10） | 1.35（1.12，1.68） | NS | 1.71（1.32，2.03） | 1.30（1.044，1.56） | 1.34（1.13，1.58） | <0.001*** |
| CD19+#, n/ul | 238.78（208.63，323.57） | 405.04（288.50，586.50） | <0.001*** | 614.60（477.10，972.46） | 448.69（306.18，667.78） | 342.75（244.50，450.44） | <0.001*** |
| IL-2, pg/ml | 5.90（5.00，6.70） | 5.59（3.39,6.45） | <0.001*** | 5.32（3.09，5.79） | 4.80（1.94，5.90） | 5.90（2.37，6.70） | NS |
| IL-4, pg/ml | 5.75（4.38，6.93） | 4.15（2.08，6.13） | NS | 3.23（0.53，6.70） | 4.45（1.88，6.40） | 4.50（2.24，6.13） | NS |
| IL-6, pg/ml | 3.4（2.6，5.00） | 5.20（3.00，8.42） | <0.001*** | 5.85（3.13，12.97） | 5.41（2.78，9.49） | 4.30（3.04，7.50） | NS |
| IL-10 | 5.3（4.28，7.03） | 5.00（3，52，7.40） | NS | 5.30（3.96，8.78） | 5.13（3.84，9.43） | 4.95（3.34，6.930 | NS |
| Interferon γ | 7.75（5.88，9.85） | 8.24（5.83，14.20） | NS | 8.96（7.00，17.01） | 8.90（6.40，21.17） | 7.80（5.45，11.00） | NS |
| Tumor necrosis factor α | 5.15（4.7，6.2） | 6.10（3.99，8.91） | NS | 6.00（4.00，8.18） | 5.63（3.78，9.55） | 5.70（3.92，8.78） | NS |
